# Circadian Dependence of the Acute Immune Response to Myocardial Infarction

**DOI:** 10.3389/fphar.2022.869512

**Published:** 2022-05-25

**Authors:** Aoife B. Kilgallen, Frederieke van den Akker, Dries A. M. Feyen, Sandra Crnko, Christian J. B. Snijders Blok, Hendrik Gremmels, Bastiaan C. du Pré, Robin Reijers, Pieter A. Doevendans, Saskia C. A. de Jager, Joost P. G. Sluijter, Vasco Sampaio-Pinto, Linda W. van Laake

**Affiliations:** ^1^ Department of Cardiology, Experimental Cardiology Laboratory, University Medical Center Utrecht, Utrecht, Netherlands; ^2^ Circulatory Health Laboratory, Regenerative Medicine Center Utrecht, University Medical Center Utrecht, Utrecht, Netherlands; ^3^ Basalt Rehabilitation, The Hague, Netherlands; ^4^ Department of Medicine and Cardiovascular Institute, Stanford University, Stanford, CA, United States; ^5^ Department of Medical Microbiology, University Medical Center Utrecht, Utrecht, Netherlands; ^6^ Division of Internal Medicine, Erasmus Medical Centre, Rotterdam, Netherlands; ^7^ Netherlands Heart Institute, Utrecht, Netherlands; ^8^ Central Military Hospital, Utrecht, Netherlands; ^9^ Utrecht University, Utrecht, Netherlands

**Keywords:** myocardial infarction, immune response, inflammation, circadian rhythms, leukocytes, neutrophils

## Abstract

Circadian rhythms influence the recruitment of immune cells and the onset of inflammation, which is pivotal in the response to ischemic cardiac injury after a myocardial infarction (MI). The hyperacute immune response that occurs within the first few hours after a MI has not yet been elucidated. Therefore, we characterized the immune response and myocardial damage 3 hours after a MI occurs over a full twenty-four-hour period to investigate the role of the circadian rhythms in this response. MI was induced at Zeitgeber Time (ZT) 2, 8, 14, and 20 by permanent ligation of the left anterior descending coronary artery. Three hours after surgery, animals were terminated and blood and hearts collected to assess the immunological status and cardiac damage. Blood leukocyte numbers varied throughout the day, peaking during the rest-phase (ZT2 and 8). Extravasation of leukocytes was more pronounced during the active-phase (ZT14 and 20) and was associated with greater chemokine release to the blood and expression of adhesion molecules in the heart. Damage to the heart, measured by Troponin-I plasma levels, was elevated during this time frame. Clock gene oscillations remained intact in both MI-induced and sham-operated mice hearts, which could explain the circadian influence of the hyperacute inflammatory response after a MI. These findings are in line with the clinical observation that patients who experience a MI early in the morning (i.e., early active phase) have worse clinical outcomes. This study provides further insight on the immune response occurring shortly after an MI, which may contribute to the development of novel and optimization of current therapeutic approaches.

## Introduction

Living organisms have evolved to efficiently interact with their surrounding environment. In particular, the time of day is a critical factor that dictates the function of organisms in nature and for which specific diurnal physiological processes are controlled by endogenous circadian rhythms (Dibner et al., 2010). These internal biological “clocks” help to anticipate daily changes in the environment (*e.g.,* temperature fluctuation) and prepare the body for behavioral changes such as prolonged periods of activity and rest (Du Pre et al., 2014; Dierickx et al., 2016). Circadian rhythms are generated when light, the main Zeitgeber (ZT; or time giver), is received in the retina and is transmitted to the master clock, the suprachiasmatic nucleus (SCN) of the hypothalamus. In turn, the SCN releases neural and hormonal signals, such as melatonin, that synchronize the peripheral clocks, consisting of tightly controlled transcriptional/translational feedback loops that cycle over 24 h and are found in almost every tissue (Crnko et al., 2019). This allows the organism to use energy supplies more efficiently by investing on processes that are crucial during a specific time period and suppressing others required at the opposing end of the circadian cycle. Nonetheless, these cyclical fluctuations to physiology influence the ability of the body to handle stress (Manfredini et al., 1998; Lee and Ederyl, 2008).

The occurrence of cardiovascular related events is influenced by the circadian rhythm, with the highest prevalence of myocardial infarction (MI) (Muller et al., 1985), sudden cardiac death (Muller et al., 1987), and arrhythmias (Portaluppi and Hermida, 2007) occurring during the rest-to-activity transition which, in humans, corresponds to the early hours of the morning. In a prospective study, patients undergoing heart surgery in the morning were reported to be at greater risk of developing major cardiac events and poorer clinical outcomes compared to patients who undergo heart surgery in the afternoon (Montaigne et al., 2018), although pooled retrospective cohort studies could not confirm this (Lecour et al., 2021). In contrast, a retrospective study analyzing the occurrence of periprocedural myocardial infarction in patients subjected to percutaneous coronary intervention (PCI), has shown an increased risk for patients undergoing PCI in the afternoon as opposed to those receiving it in the morning (Fournier et al., 2014). Both murine (Durgan et al., 2010) and several clinical studies (Suarez-Barrientos et al., 2011; Fournier et al., 2012), concluded that myocardial damage is greater when a MI occurs during the sleep-to-wake transition period, demonstrated by the development of larger infarct sizes and higher levels of creatinine kinase release. Although studies performed in cardiomyocyte-specific circadian clock mutant mice have suggested that cardiomyocytes are more vulnerable to MI-induced stress during this transition period (Durgan et al., 2010), additional mechanisms underpinning these findings remain unclear.

A poorly studied aspect in this phenomenon is the immune system, which plays a major role in the pathophysiology of heart failure. During a MI, immune cells are recruited to the injury site in order to clear dead cells and debris (Frangogiannis et al., 2002; Kaminski et al., 2002). This process promotes healing after injury, leading to scar formation and adaptive remodelling of the heart to preserve cardiac function. Although inflammation is essential for cardiac repair, an exacerbated immune response can lead to additional damage to the heart (Kaminski et al., 2002). Immune cells involved in the innate immune response, called “early-responders”, are hyper-reactive during the ischemia and reperfusion which triggers the production of factors that worsen myocardial injury (Kaminski et al., 2002; Ungvari et al., 2005; Epelman et al., 2015).

In humans and mice, the circulation and infiltration of immune cells and its upstream mediators (cytokines, chemokines) display time-dependent oscillations in both tissue and blood (Scheiermann et al., 2013). The oscillatory pattern in the recruitment of neutrophils may be responsible for the circadian-dependent damage of the myocardium after a MI (Muller et al., 1985; Suarez-Barrientos et al., 2011). Patients with higher neutrophil and leukocyte counts were found to develop larger infarcts and suffer more serious adverse cardiac events (Chia et al., 2009). Furthermore, in a murine model of ischemia/reperfusion, Schloss and colleagues demonstrated a preferential recruitment and infiltration of neutrophils to the heart during the active phase at ZT13, correlating with worse outcomes, 24 h after induction of a MI (Schloss et al., 2016). In the clinical setting, however, the median time frame for MI patients to receive primary PCI is approximately 180 min post-MI (Nallamothu et al., 2005). The immune system is so dynamic throughout the day, in both physiological and pathophysiological settings, that studying the immune response 24 h post-MI, may not give an accurate representation of the inflammatory response occurring when patients are treated for a MI. Hence, in this study, we characterized the circadian-dependent response of the immune system 3 hours after a MI occurs, over a 24-h period. Exploring the hyperacute immune response in depth, in this clinically relevant time frame, may provide additional opportunities to develop therapeutic interventions that can effectively target the immune response after a MI.

## Materials and Methods

### Animal Experiments

All experiments were carried out in accordance with the *Guide for the Care and Use of Laboratory Animals*, with prior approval by the Animal Ethical Experimentation Committee, Utrecht University. Male Balb/c mice (Jackson), aged 10–12 weeks were housed under controlled conditions in a 12-h light/12-h dark cycle (lights on at ZT0, lights off at ZT12). Water and food were provided *ad libitum*. Mice were anesthetized by intraperitoneal injection (fentanyl 0.05 mg/kg; midazolam 5 mg/kg; medetomidine 0.5 mg/kg) and subjected to MI by LAD coronary artery ligation or to sham surgery, as previously described (Van Laake et al., 2007), at 4 different timepoints (ZT2, ZT8, ZT14, and ZT20). To avoid disturbances to their circadian rhythm by light, animals undergoing surgery in periods of darkness were subjected to anesthesia while still in their light cycle room and transported to the operating room after sedation. Before any incision was made, the adequacy of anesthesia was monitored by testing rear foot reflexes. Continual observation of the respiratory pattern, rectal temperature, and responsiveness to manipulations was carried out throughout the procedure. Baseline blood samples were collected via tail clipping prior to the start of surgery. After finalizing surgery, animals were immediately placed in their original housing conditions and were euthanized 3 hours after surgery. Blood was collected via the retro-orbital sinus, placed in EDTA tubes and used for flow-cytometric analysis. Plasma was separated from the blood and stored at −80°C. Hearts were harvested and cut longitudinally. One half was inserted in paraffin for histology, while the other was snap frozen and stored at −80°C until further analysis (i.e., real-time quantitative polymerase chain reaction, RT-qPCR). An illustration of the experimental design can be found in the Supplementary Figure S1.

### Flow Cytometry

Blood cells from whole blood were incubated with fluorochrome-conjugated antibodies for 30 min at room temperature. After incubation, samples were washed with PBS containing 5% FBS, after which Optilyse C (Beckman Coulter, A11895) was added to lyse erythrocytes. Antibodies against the following proteins were used: CD3e-PE (Clone 145-2C11; 12-0031-82), CD8a-APC-eFluor780 (Clone: 53-6.7; 47-0081-82), CD11b-AlexaFluor488 (Clone: M1/70; 53-0112-82), CD19-eFluor 450 (Clone: 1D3; 48-0193-82) and F4/80-PE-Cy7 (Clone: BM8; 25-4801-82) purchased from eBioscience. From BD Bioscience CD4-PerCP (Clone RM4-5; 553,052), and Ly6G-APC (Clone 1A8; 560599) were used. The Gallios Flow Cytometer (Beckman Coulter) was used to measure cell fluorescence and all analyses were performed with Kaluza Analysis Software (Beckman Coulter, version 1.3). Results are expressed as the number of immune cells per milliliter. To determine this concentration, the number of cells determined in a fixed volume of blood (20 μL at baseline and 50 μL at termination) was multiplied by 50 and 20, respectively, to estimate the number of cells in 1 mL. The gating strategy can be found in the Supplementary Figure S2.

### Plasma Measurements

High-sensitive Troponin I was measured in the collected plasma using a clinical chemistry analyzer (AU5811, Beckman Coulter). The plasma levels of inflammatory cytokines and chemokines were determined using a 36-multiplex panel (eBioscience, EPX360-26092-01), measured with a Luminex-200 instrument (Bio-Plex 200). The Luminex assay was performed according to manufacturer’s protocol.

### Histological Analysis

Upon termination, hearts were excised and fixed in 4% formaldehyde and processed for paraffin embedding. Paraffin sections were deparaffinized and stained for Ly6G (1:100—Abcam, ab210204), Mac-3 (1:30—BD Pharmingen, 553322) and CD3 (1:100—eBioscience, 14-0032-81). After incubation with alkaline phosphatase conjugated secondary antibody, staining was visualized with Liquid Permanent Red substrate kit following the manufacturer’s instructions (DAKO). All sections were counterstained with Mayer’s hematoxylin stain.

### RNA Isolation and Real-Time Polymerase Chain Reaction (RT-qPCR)

RT-qPCR was performed to investigate the genetic expression of *Cxcl1*, *Cxcl2*, *Icam1*, *Vcam1* and clock genes *Bmal1*, *Per1*, *Cry2*, *Clock*, *Rev-Erbα*, *Rorα,* and *Sirt1* (Table 1). Gene expression was normalized to that of the house-keeping gene *Rplp0*. Total RNA was isolated from snap frozen heart fragments using the Tripure^TM^ Isolation Reagent (Roche, 11667165001) according to the manufacturers’ protocol. After DNAse treatment, 500 ng of total RNA was used for cDNA synthesis using the iScript^TM^ cDNA synthesis kit (Bio-Rad, 1708891). RT-qPCR were performed using iQ^TM^ SYBR Green supermix (Bio-Rad, 1708880).

**TABLE 1:** List of RT-qPCR primers.

Gene	Forward primer sequence (5′→3′)	Reverse primer sequence (5′→3′)
*Cxcl1*	ATGAGCTGCGCTGTCAGTGC	CACCAGACGGTGCCATCAGA
*Cxcl2*	GCGTCACACTCAAGCTCTG	GCGCTGTCAATGCCTGAAGA
*Icam1*	CAGTGAGGAGGTGAAT GTATAAG	GATGTGGAGGAGCAGAGAAC
*Vcam1*	CACCCTCACCTTAATTGCTATG	CGTCAGAACAACCGAATCC
*Rplp0*	GGACCCGAGAAGACCTCCTT	GCACATCACTCAGAATTTCAATGG
*Bmal1*	TGACCCTCATGGAAGGTTAGGTTAGAA	GGACATTGCATTGCATGTTGG
*Per1*	TCGAAACCAGGACACCTTCTCT	GGGCACCCCGAAACACA
*Rev-Erbα*	CGTTCGCATCAATCGCAACC	GATGTGGAGTAGGTGAGGTC
*Rorα*	GTGGACATTGGCATGATGAAGG	CATCGAGCAGGATGGCGATA
*Clock*	AAAGACGGCGAGAACTTGG	GAAGGCAGAAGGAGTTGGG
*Cry2*	CCTCGTCTGTGGGCATCAA	GCTTTCTTAAGCTTGTGTCCAGATC
*Sirt1*	CGGCAGCGATCGGCTA	TTAGTGAGGAGTCCATCGGTCA

### Statistical Analysis

All statistical analyses were performed in SPSS statistics 20 (IBM, Armonk, NY). Data were tested using a one-way ANOVA with a LSD post-hoc test. 24-h rhythmicity was analyzed using the online platform Cosinor. Online (https://cosinor.online/app/cosinor.php). Error bars represent standard error of mean (SEM). A *p*-value below 0.05 was considered significant.

## Results

### Steady-State Oscillations of Leukocytes Occur in the Blood of Healthy Mice

**FIGURE 1:**
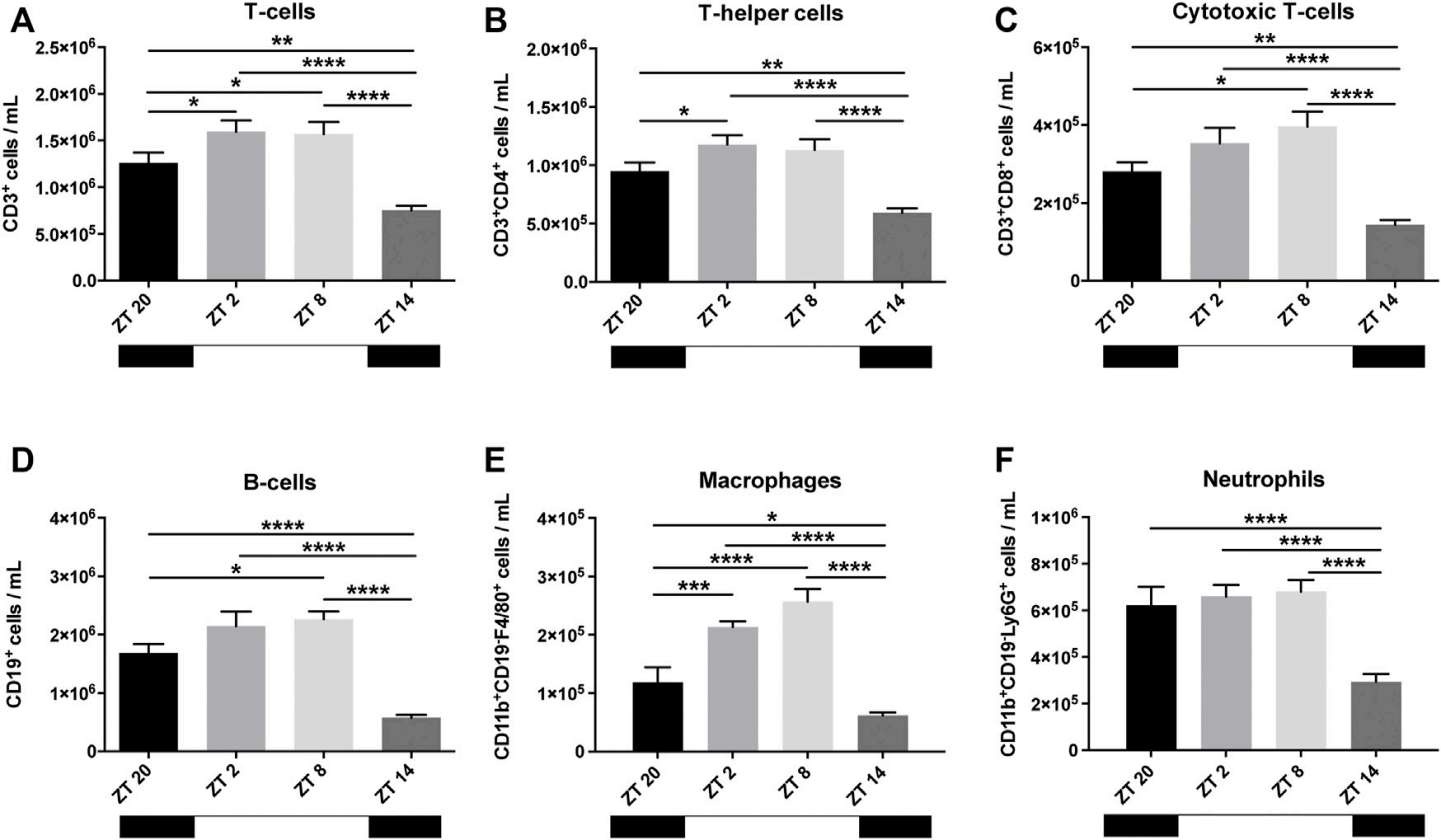
Baseline leukocyte count in the blood. T-cells **(A)**, further subdivided in helper T-cells (T_H_-cells) **(B)** and cytotoxic T-cells (T_C_-cells) **(C)** showed an oscillating pattern, reaching its highest frequency at ZT2-ZT8 (T_H_-cells) and ZT8 (T_C_-cells). B-cells also had a clear oscillation, reaching highest circulatory levels at ZT2-ZT8 **(D)**. Macrophages **(E)** showed a pattern similar to B- and T-cells. Neutrophils had stable high levels, which dropped at ZT14 **(F)** (*n* = 10–12 per group for all analysis) (*p < 0.05, **p < 0.01, ***p < 0.001, and ****p < 0.0001 by one-way ANOVA with LSD test).

To investigate the existence of baseline circadian patterns in the circulation of leukocytes, blood was collected from mice before MI-induction or sham surgery at different time points (ZT2, ZT8, ZT14, and ZT20). CD3^+^ T-cells, which were further subdivided into CD4^+^ T-helper cells (T_H_-cells) and CD8^+^ cytotoxic T-cells (T_C_-cells), were less frequent at ZT14 (Figures 1A–C). While CD4^+^ T_H_-cells were particularly prevalent at ZT2-ZT8 (Figure 1B), CD8^+^ T_C_-cells peaked at ZT8 (Figure 1C). The number of circulating CD19^+^ B-cells was also lowest at ZT14, with the highest cell count peaking at ZT2-ZT8 (Figure 1D). The oscillatory nature of macrophages (CD11b^+^CD19^-^F4/80^+^) in blood was more pronounced but in line with the previous cell types, peaking at ZT8 and rapidly decreasing at ZT14 (Figure 1E). On the contrary, the number of circulating neutrophils (CD11b^+^CD19^−^Ly6G^+^) was found to be rather stable from ZT20-ZT8 but showed a significant reduction at ZT14 (Figure 1F). Altogether, these results indicate that during the rest phase (i.e. ZT2-ZT8), mice have higher levels of circulating leukocytes in comparison to the active phase (i.e., ZT14-ZT20).

### Leukocytes in the Blood After MI Display a Circadian Pattern

**FIGURE 2:**
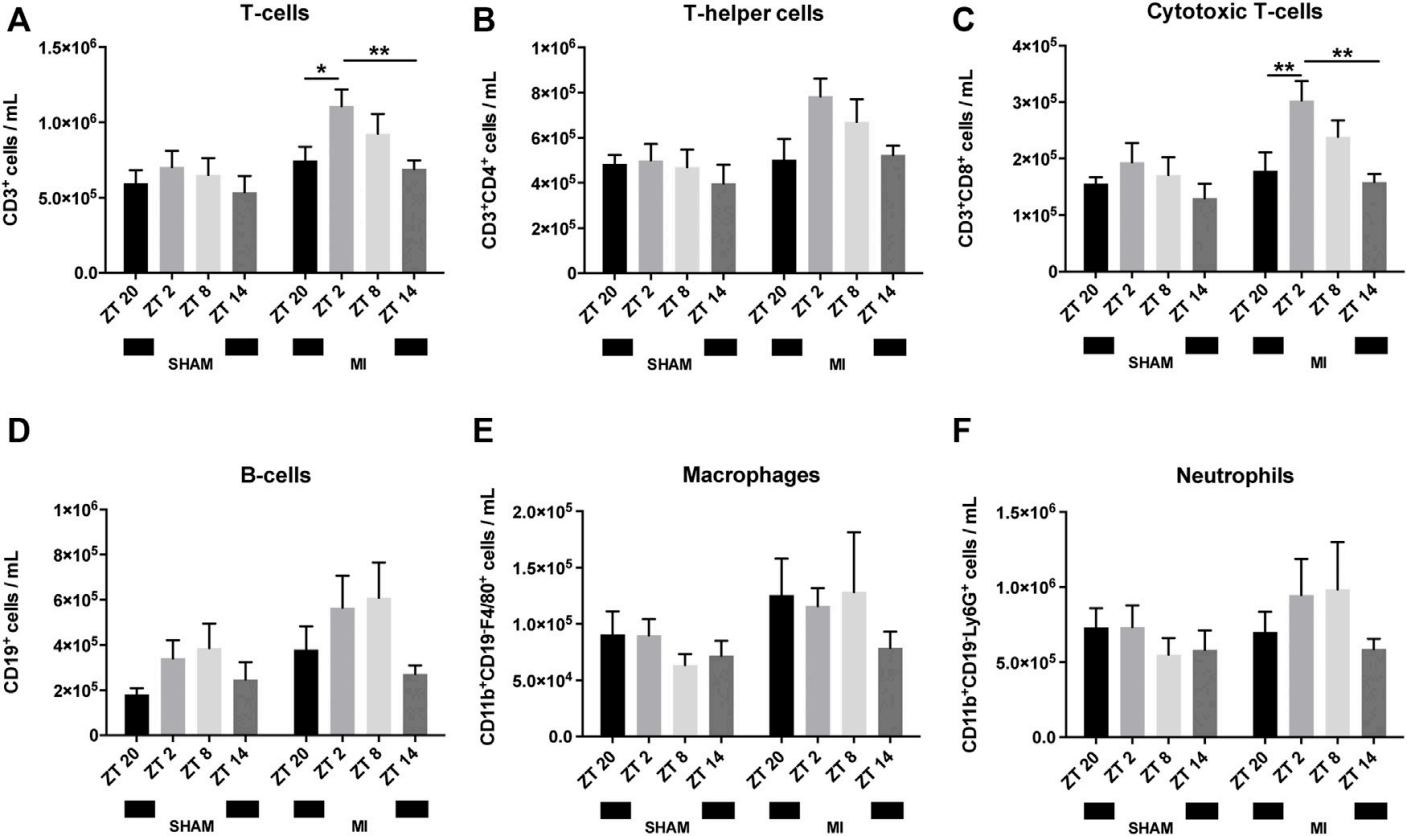
Leukocyte numbers in blood after surgery. Number of circulating T-cells **(A)**, T_H_-cells **(B)**, T_C_-cells **(C)**, B-cells **(D)**, macrophages **(E)** and neutrophils **(F)** (*n* = 5–6 per group for all analysis) (*p < 0.05, **p < 0.01 by one-way ANOVA with LSD test).

We next investigated if similar circadian patterns also occur post-surgery. To study this, MI or sham surgery was performed at ZT2, ZT8, ZT14 or ZT20. Mice were terminated 3 hours after surgery and their blood collected. Animals subjected to MI at ZT2 and ZT8 had consistently higher levels of circulating leukocytes, in comparison to mice subjected to MI at ZT14 or ZT20, with the exception of CD11b^+^CD19^−^F4/80^+^ macrophages which were comparably prevalent at ZT20, ZT2 and ZT8 (Figures 2A–F; Supplementary Figures S3–S5). Yet, this increase only reached statistical significance for CD3^+^ T-cells, predominantly CD8^+^ T_C_-cells, which peaked at ZT2 (Figures 2A,C). Sham-operated mice had a similar trend (i.e., increased number of circulating leukocytes at ZT2 and ZT8) but no significant differences were detected in any of the immune subsets (Figures 2A–F).

The baseline circadian pattern observed in CD8^+^ T_C_-cells (Figure 1C) became less pronounced after MI or sham-surgery, with the highest cell counts occurring in the animals undergoing surgery at ZT2 (Figure 2C).

In regards to CD11b^+^CD19^-^F4/80^+^ macrophages, the oscillatory pattern visible at baseline (Figure 1E) was completely disrupted post-MI or sham surgery (Figure 2E). High levels of circulating CD11b^+^CD19^-^F4/80^+^ macrophages were detected from ZT20 to ZT8, while its lowest levels were observed at ZT14 in the MI group (Figure 2E). In sham-operated mice, circulating CD11b^+^CD19^-^F4/80^+^ macrophages appeared in relatively low numbers in all of the timepoints assessed, reaching a minimum in animals subjected to surgery at ZT8 (Figure 2E).

Concerning CD11b^+^CD19^−^Ly6G^+^ neutrophils, the oscillatory pattern observed at baseline was also disrupted after MI or sham-surgery. In contrast to the baseline measurements (Figure 1F), the number of circulating neutrophils in MI mice was reduced at ZT20, leading to a diphasic pattern with low neutrophil numbers from ZT14-ZT20, and elevated numbers from ZT2-ZT8 (Figure 2F). In turn, in sham-operated mice, we observed that the minimum number of circulating neutrophils was achieved when the surgery was carried out at ZT8 (Figure 2F).

### Leukocyte Invasion Into the Myocardium After a MI

**FIGURE 3:**
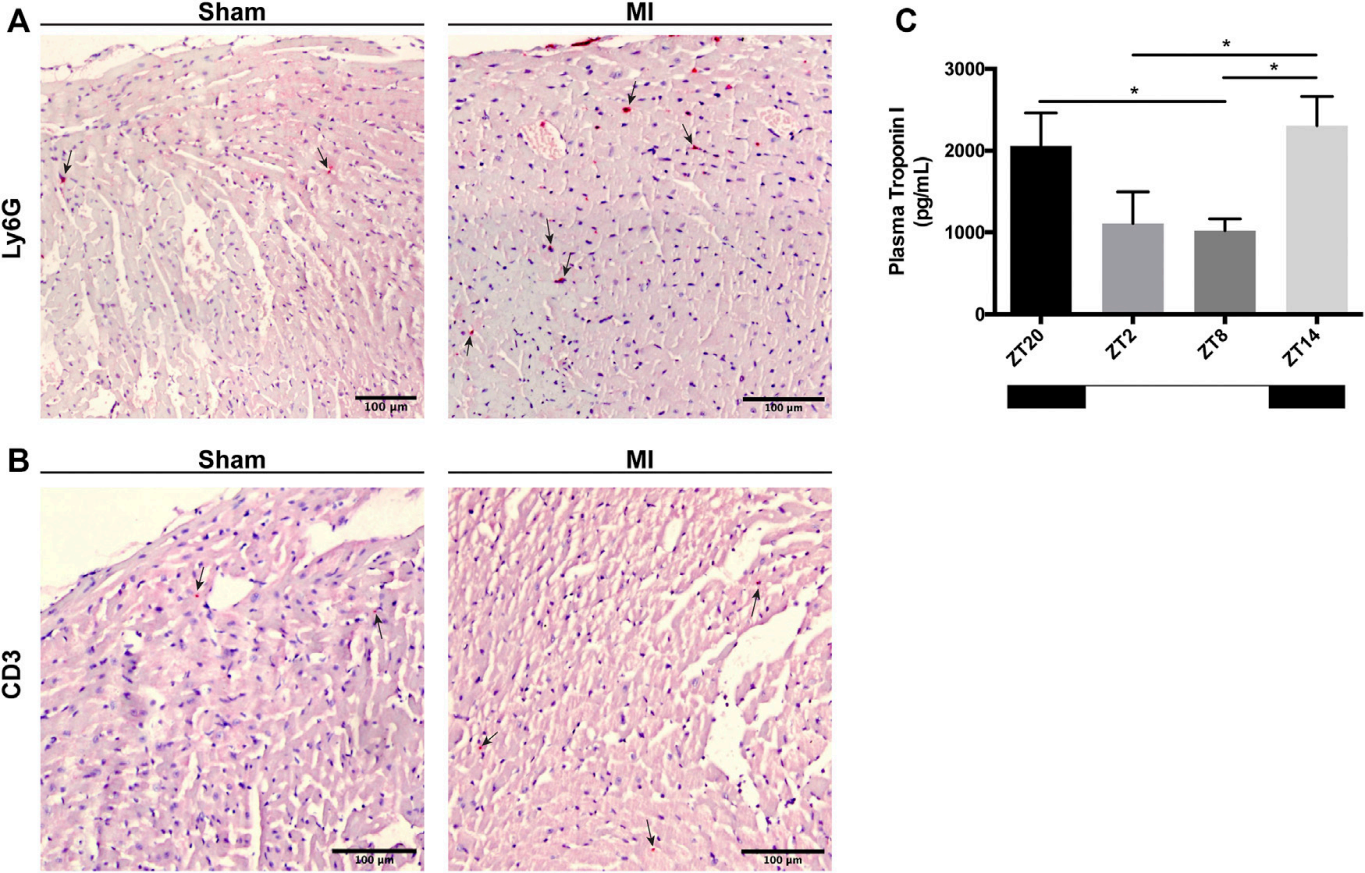
Immune cell infiltration in the heart and cardiac damage 3 h after MI induction. **(A)** Representative images showing Ly6G^+^ neutrophils in the myocardium (black arrows; scale bar: 100 μm). **(B)** Representative images showing CD3^+^ T-cells in the myocardium (*black arrows;* scale bar: 100 µm). **(C)** Troponin-I levels in the plasma of mice subjected to MI at ZT2, ZT8, ZT14 and ZT20, 3 h after injury. (*n* = 5–6 per group for all experiments) (*p < 0.05 by one-way ANOVA with LSD test).

Next, we explored whether there is a circadian influence in the recruitment of leukocytes into the myocardial tissue after MI induction. In heart sections, we screened for the presence of different immune cell types that have been implicated in the acute response to myocardial injury including neutrophils (Ly6G^+^), macrophages (Mac-3^+^) and T-cells (CD3^+^). In both sham- and MI-induced hearts, we were unable to detect the presence of Mac-3^+^ macrophages (data not shown), indicating that these cells do not play a significant role in such an early stage of response to injury, *i.e,.* 0–3 h post-MI. In contrast, Ly6G^+^ neutrophils and CD3^+^ T-cells were observed in the hearts subjected to surgery, particularly in the areas in proximity to the site of coronary ligation in MI-induced hearts, and independently of the time of day the surgery took place (representative images in Figures 3A,B). Plasma troponin I was found to be greatest in mice subjected to MI at ZT14, indicating that myocardial damage is potentiated when MI takes place during the early-active phase (Figure 3C).

### Chemokine Oscillation in the Blood After a MI

**FIGURE 4:**
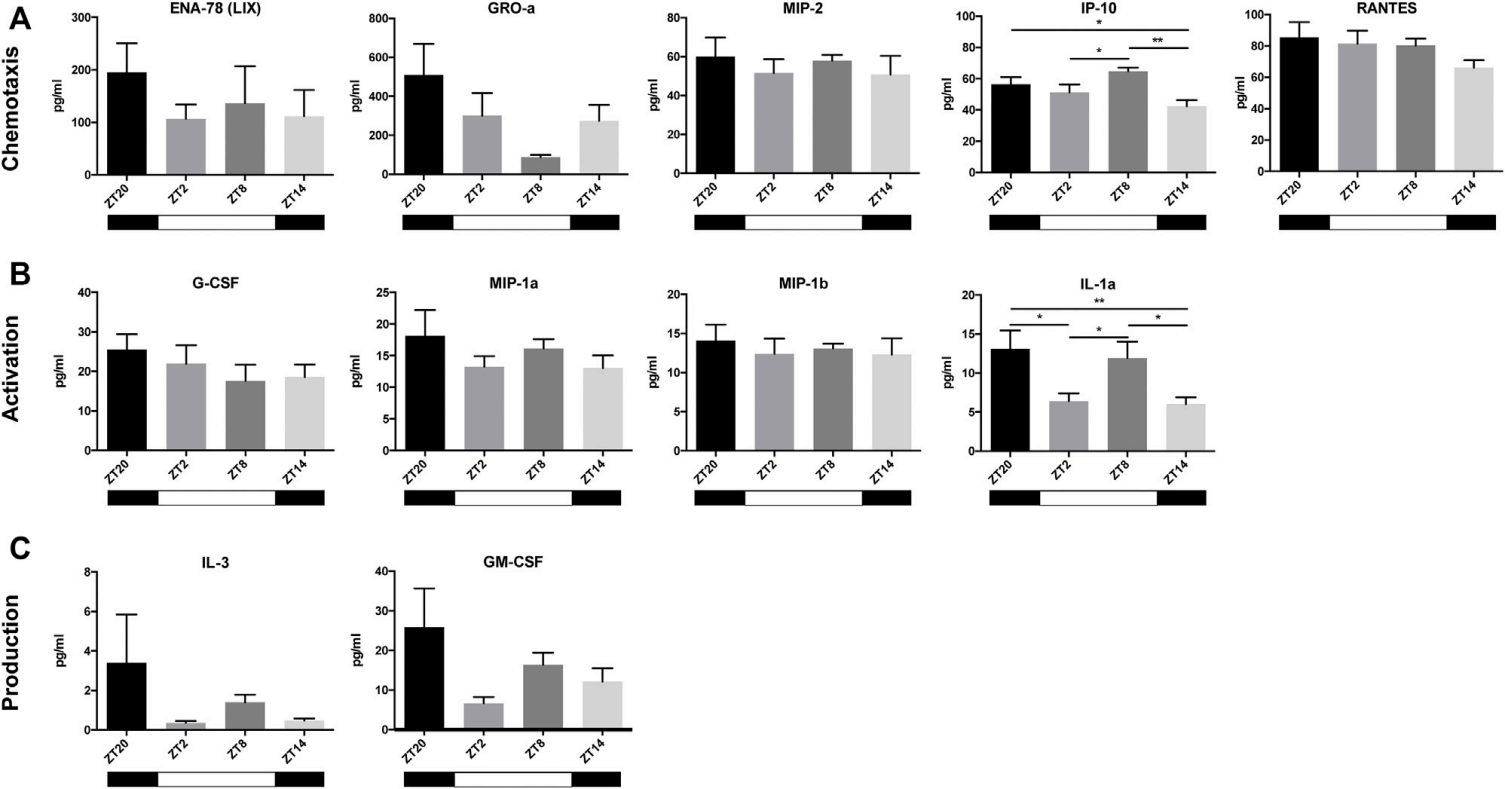
Cytokines and chemokines in the blood after MI. Most chemokines involved in the chemotaxis of neutrophils (ENA-78, GRO-a, MIP-2, and RANTES) reached the highest plasma concentrations at ZT20, with the exception of IP-10, which peaked at ZT8 **(A)** Cytokines involved in neutrophil activation (G-CSF, MIP-1a, MIP-1b, and IL-1alpha) all peaked at ZT20 **(B)** Cytokines that play a role in the stimulation of neutrophil production and release (IL-3 and GM-CSF) peaked at ZT20 **(C)** (*n* = 5–6 per group for all analysis) (*p < *0.05*, **p < 0.01 by one-way ANOVA with LSD test).

Next, given the widely-recognized role of neutrophils in the acute response after MI, we measured the plasma levels of neutrophil-related cytokines and chemokines, 3 h post-MI, at different timepoints, to investigate if their expression in the blood also varies in a circadian dependent manner. Cytokines were subdivided into three neutrophil functional groups: 1) chemotaxis, 2) activation and 3) production. We found a tendency for cytokines associated with neutrophil chemotaxis to show their highest concentration at ZT20, with the exception of IP-10 which peaked at ZT8 (Figure 4A). A non-significant similar pattern was visible in cytokines involved in neutrophil activation or degranulation, which reached their maximum concentrations at ZT20. In contrast, IL-1alpha expression was found to be more variable, with higher concentrations being found when MI was induced at ZT20 and ZT8. The lowest concentration of these cytokines occurred predominantly at ZT14 (Figure 4B). Likewise, cytokines stimulating the differentiation of new neutrophils (*i.e.* IL-3 and GM-CSF) showed highest plasma levels at ZT20. Hence, these results indicate that both myocardial damage (Figure 3C) and neutrophil activity (Figure 4) are maximized when an MI occurs during the active phase (ZT14-ZT20).

### Chemokine and Adhesion Molecule Expression in the Heart

**FIGURE 5:**
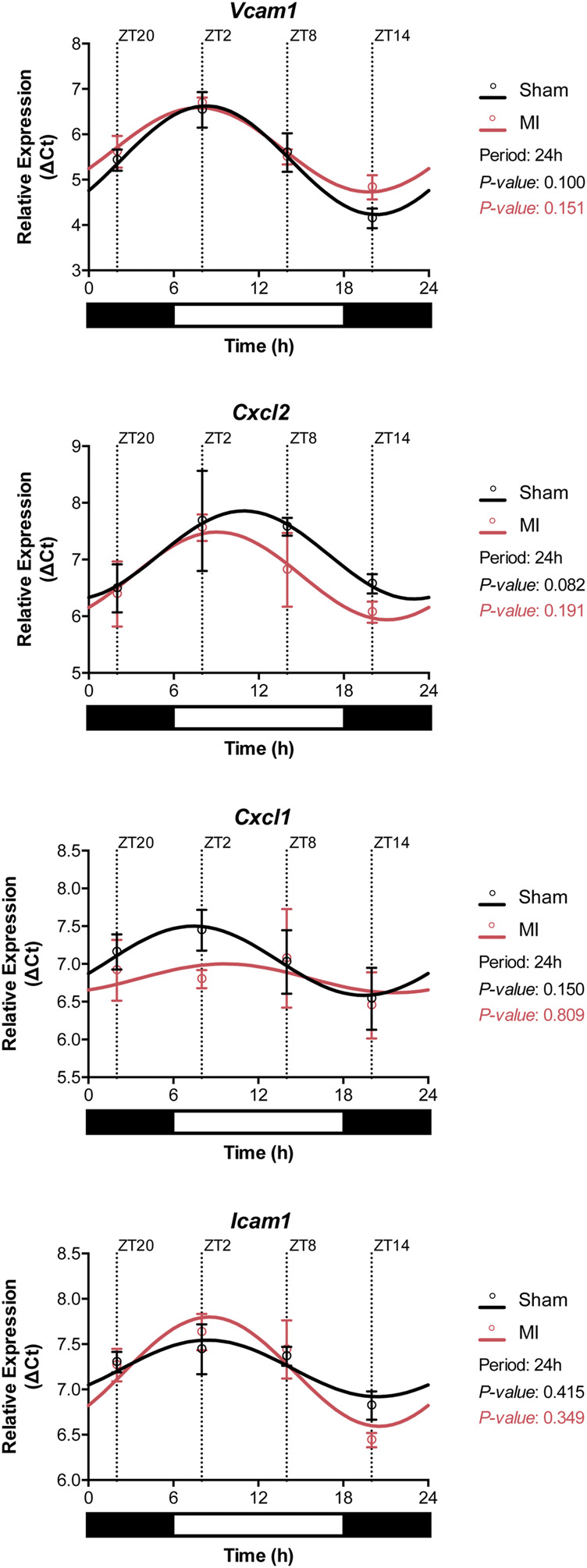
Gene expression of adhesion molecules in the heart. The expression of *Vcam1*, *Cxcl2, Cxcl1,* and *Icam1* followed circadian-like oscillations with higher levels present during the active phase (ZT14-ZT20). (*n* = 3 per group/timepoint for all analysis, *p*-values determined by Cosinor analysis using the online platform: Cosinor. Online (https://cosinor.online/app/cosinor.php)).

The infiltration of leukocytes from the blood into the myocardial tissue is known to be regulated by the local cardiac expression of chemotactic gradients and adhesion molecules (Frangogiannis, 2014). Analysis of gene expression in heart samples from sham and MI mice showed that *Vcam1*, *Cxcl2, Cxcl1,* and *Icam1* display a circadian-like oscillatory pattern (Figure 5). The highest expression of these genes was achieved during the active period (*i.e,.* ZT14) while downregulation of the aforementioned genes was observed during rest-phase (*i.e.,* ZT2-ZT8) (Figure 5; Supplementary Table S1).

### Clock Gene Oscillation Remains Intact in Heart Tissue After a MI

**FIGURE 6:**
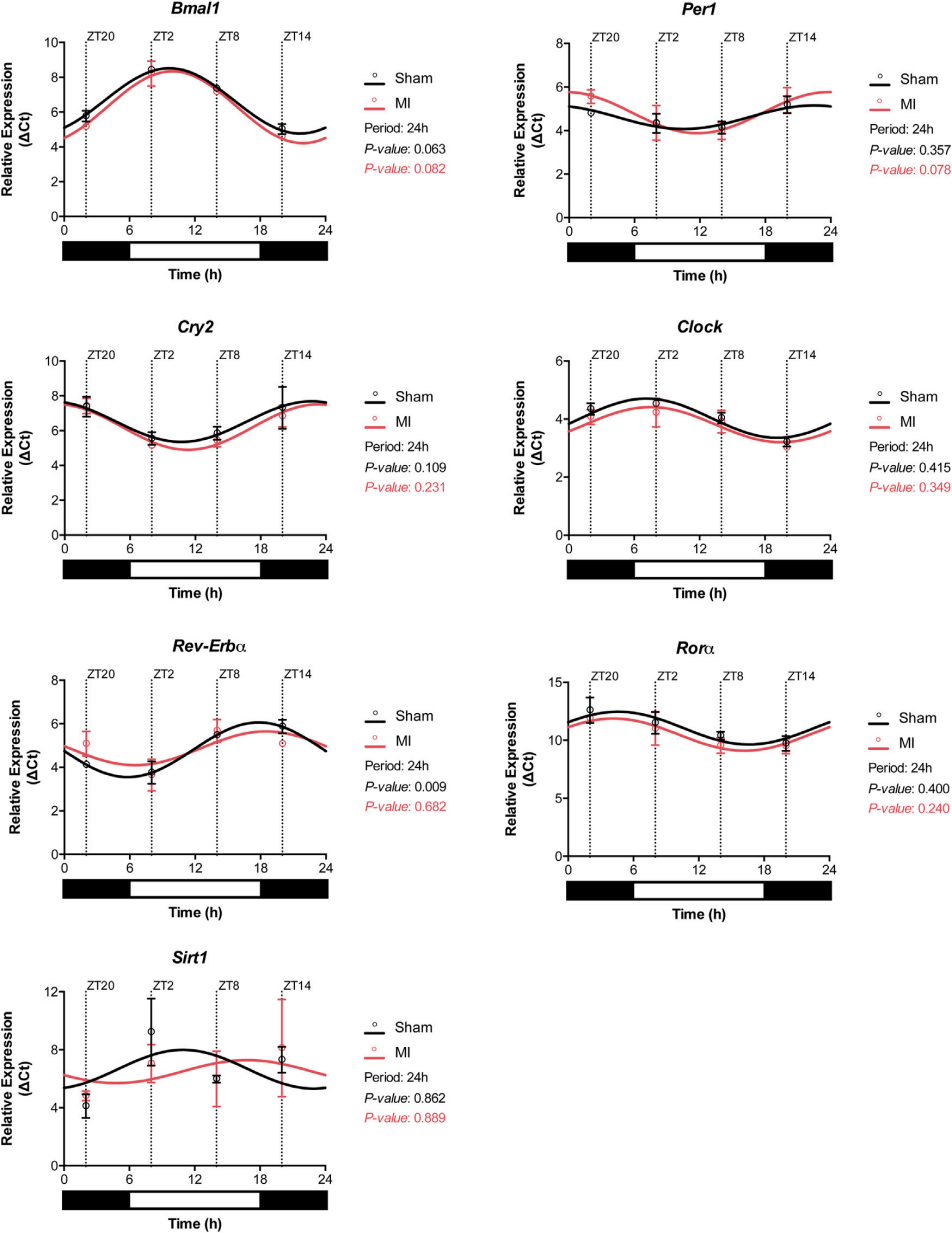
Circadian clock gene expression profile in the heart. Expression of the circadian clock genes *Bmal1*, *Per1*, *Cry2*, *Clock*, *Rorα*, *Rev-Erbα*, and *Sirt1* in the heart of sham- and MI-induced mice. (*n* = 3 per group/timepoint for all analysis, (*p < 0.05, **p < 0.01, and ***p < 0.001, *p*-values determined by Cosinor analysis using the online platform: Cosinor. Online (https://cosinor.online/app/cosinor.php)).

Circadian oscillations in gene expression are typically regulated by upstream expression of clock genes, subjected themselves to synchronization by external and internal factors. To determine whether this machinery is intact or disrupted post-MI, we evaluated the expression of clock genes in the heart after sham or MI surgery at different time points. All of the analyzed clock genes exhibited an oscillatory expression in both sham-operated and MI-induced hearts suggesting that MI does not affect substantially the circadian expression of clock genes (Figure 6; Supplementary Table S1). Interestingly, *Sirt1* expression was found to be slightly off-phase in MI-induced hearts in comparison to sham-controls.

## Discussion

The time of day affects the clinical outcome of patients that suffer a MI (Suarez-Barrientos et al., 2011). Although previous studies primarily focused on cardiomyocytes, the immune system is known to play a very important role in the pathophysiology post-MI, including heart failure, and has been shown to be robustly controlled by circadian rhythms (Scheiermann et al., 2013; Labrecque and Cermakian, 2015). Schloss et al. confirmed that the immune system, in particular neutrophil recruitment to the infarcted heart, was associated with the development of larger infarct sizes 24 h after MI induction, particularly during the active phase at ZT13 (Schloss et al., 2016). In a clinical setting, however, therapeutic interventions are administered to patients within a 3-h period after a MI occurs (Nallamothu et al., 2005). The immune response after a MI is fast, dynamic, and progressive. Given the lack of knowledge regarding the hyperacute immune response after MI, in the present study, we aimed to investigate the circadian oscillation of the immune cells, cytokines and chemokines and their association with myocardial damage within the first 3 h after MI induction. Almost every aspect of the immune system, including immune cell mobilization, is tightly controlled by the circadian clock (Orozco-Solis and Aguilar-Arnal, 2020). For example, in both mice (Scheiermann et al., 2012) and humans, the number of neutrophils, macrophages and leukocytes in the blood oscillate throughout the day (Aroca-Crevillen et al., 2020). From the blood samples collected at baseline, we observed strong oscillations in blood leukocyte numbers that followed a strict rest-to-activity pattern (Figures 1A–F). High levels of circulating immune cells were detected during periods of rest, while a strong reduction was observed at the onset of the active period, which is in line with previous reports (Haus et al., 1983; Haus and Smolensky, 1999). After surgery, these oscillations were less evident, especially in the sham-treated animals (Figure 2). This suppression is likely due to the invasive thoracotomy operation, which in itself, causes local and systemic effects (Mitsos et al., 2009). Mice that underwent LAD ligation displayed a propensity to have more circulating immune cells (B-cells, T-cells, and phagocytes) in comparison to sham-operated animals (Figure 2). Furthermore, the response of T-cells and their different subpopulations in the MI-induced group followed a circadian oscillation similar to that observed before the surgery, with higher numbers during the rest-phase and a sharp decrease upon entering the active period (Figure 2). These oscillatory patterns were however disturbed in macrophages and neutrophils post-MI, which may be explained by alterations to the circadian clock network, in particular *Sirt1* expression, which is shown to be downregulated in murine models of ischemia/reperfusion after a MI (Yang et al., 2013; Wang et al., 2018). Importantly, as demonstrated in the publication by Hoggatt et al., the bleeding method can affect the estimation of white blood cells in circulation (Hoggatt et al., 2016), which can also partially explain the differences between our baseline and post-surgery measurements.

Immunolabelling of inflammatory cells in heart tissue sections revealed that CD3^+^ T-cells and Ly6G^+^ neutrophils accumulate in the myocardium 3 h after surgery (Figures 3A,B). In a previous publication (Schloss et al., 2016), the infiltration of leukocytes in the myocardium was reported to follow a diurnal rhythm with an opposing phase to that observed in the blood. In the hyperacute response to MI studied herein, we did not observe a time-dependent accumulation of leukocytes in the myocardium. However, we did detect a decrease in leukocyte numbers in the blood during the active phase (ZT14-ZT20) and when the expression of adhesion molecules in the heart was highest (Figures 2, 5). Our results are in agreement with other studies showing that the heart is most susceptible to myocardial injury during the active phase of the circadian cycle (Suarez-Barrientos et al., 2011; Fournier et al., 2012). In our setting, plasma troponin I levels were increased by 2-fold when the ligation was placed during the active period of the day (ZT14 and ZT20) (Figure 3C). While some evidence exists for the presence of diurnal rhythms in the concentration of cardiac troponin T in healthy volunteers (Fournier et al., 2017), a large cohort study with patients with chest pain but no MI nor acute condition that would justify increased troponin levels, found no evidence of diurnal variation in cardiac troponin T concentrations (Roos and Holzmann, 2018). In addition, a clinical trial evaluating the diagnostic accuracy of cardiac troponin I for acute myocardial infarction, found no differences in the levels of troponin I between patients admitted in the morning or in the evening (Wildi et al., 2018). As such, it is likely that the increased troponin I levels reported in our study are caused by myocardial damage and not due to a daily fluctuation. Importantly, neutrophil recruitment to the myocardium after MI has been associated with the development of larger infarcts, and worsened clinical outcomes (Chia et al., 2009). Conversely, limiting neutrophilic inflammation in mice proved protective for the MI-induced heart, which developed smaller infarcts and had a better-preserved systolic function (Schloss et al., 2016). Circadian rhythms also play a role in the oscillation of cytokines and chemokines which, in turn, regulate immune cell mobilization (Scheiermann et al., 2012). To better understand if there was a link between neutrophil numbers in the blood and the presence of factors promoting neutrophil recruitment, we harvested and screened the plasma of mice subjected to MI. We found chemokines involved in neutrophil chemotaxis and activation to be upregulated primarily in the blood of mice undergoing surgery at the early active phase (ZT14-ZT20; Figures 4A,B), which was concomitant with decreased circulating neutrophils in blood (Figure 2). Similarly, it was reported that adhesion molecules *Icam1* and *Vcam1* are highly expressed during the active phase, which can potentially explain why there is enhanced recruitment of neutrophils to the myocardium in the active phase (Schloss et al., 2016). In the study herein, we have screened for factors regulating neutrophil function in the short-term response to MI, but future work should explore the presence, involvement and molecular signature of additional immune subsets, including Ly6C^+^ circulating monocytes, only partially detected with the gating strategy employed in our study.

To understand whether the circadian clock machinery was preserved, which could explain the oscillatory patterns in the immune response detailed above, we assessed the expression of core clock genes throughout the day in sham-operated and in MI-induced hearts. In both groups, we found that most of the clock genes had a preserved expression profile with circadian-like oscillations. This could explain why the oscillation of the different components of the immune system remained intact in both MI and sham-operated mice. Nonetheless, it is important to note that by focusing on the hyperacute response to MI, this study might miss changes in circadian gene expression that occur at a later point in time.

Even though the study herein focuses on the circadian dependency of the hyperacute immune response to MI, it is worth mentioning that early differences in myocyte death during a MI can impact on the subsequent immune response. Wild-type mice and humans develop larger infarcts when MI occurs in the early active phase (Durgan et al., 2010; Arroyo Úcar et al., 2012). In contrast, clock-mutant mice develop smaller infarcts, independently of the time-of-day MI was induced, presumably due to increased activation of cardiomyocyte survival pathways (Durgan et al., 2010). While it is important to study and manage the inflammation after MI, an equally valid approach is to monitor the circadian-dependent susceptibility of cardiomyocytes towards ischemia and prevent cardiomyocyte death.

### Implication for the Clinic

The adverse inflammatory response after MI can be partially attributed to biological day/night rhythms that influence the ability of the heart to attract immune cells to the site of injury. Several studies resorting to general immune suppressive therapy during or after MI have had disappointing results (reviewed in (Huang and Frangogiannis, 2018)). Moreover, general immunosuppression using NSAIDs appeared to increase the incidence of cardiac rupture (Brown et al., 1983; Gislason et al., 2006). Importantly, the inclusion criteria of such studies fail to segregate patients undergoing MI in the active- or rest-phase of the circadian cycle, thus undermining potential benefits in a time-dependent subgroup (Gislason et al., 2006; Garcia-Prieto et al., 2017). Targeting the circadian rhythm within this time frame, for example SIRT1, may be a potential therapeutic target within the 3-h time frame. It has been reported previously that lumbrokinase, known to upregulate SIRT1 levels, can protect the hearts of rats against myocardial ischemic injury, by minimizing infarct size as well as reducing oxidative damage and inflammation (Yamamoto and Sadoshima, 2011; Wang et al., 2018). It is also important to emphasize the importance of regulating the external environment to reduce adverse clinical events in MI patients. Short term disruption of light in mice subjected to MI contributed to the development of a stronger immune response, altered immune cell infiltration and larger infarct size 5 days after a MI (Alibhai et al., 2014). Hence, it is reasonable to speculate that reducing environmental stressors, by implementing circadian lights in hospitals, could promote an improved healing of the myocardium after MI (Alibhai et al., 2014).

**FIGURE 7:**
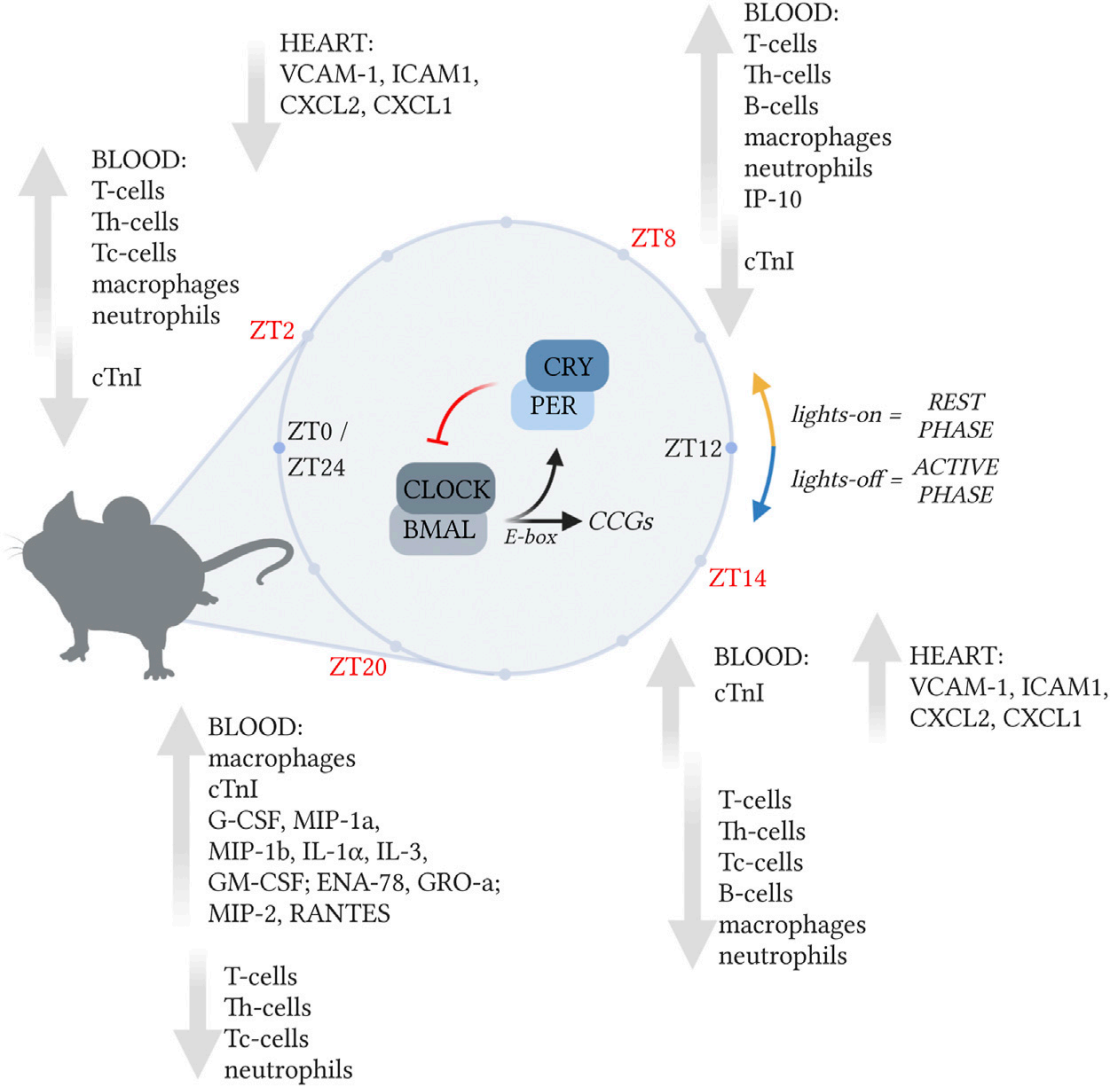
The immune response post-MI is regulated in a circadian-dependent manner. The immune response in the post-MI heart varies depending on the time of day MI occurred. In the blood, leukocyte numbers peaked during the rest-phase (ZT2 and ZT8), when chemokine and cytokine levels were low. Neutrophil-associated chemokines and cytokines reached their highest levels during the active-phase (ZT14). In the heart, the expression of chemokine and adhesion molecules (*Cxcl1*, *Cxcl2*, *Vcam1,* and *Icam1*), was maximum when MI was induced during the active-phase (ZT14). Created with BioRender.com.

In conclusion, our data suggests that myocardial damage caused by the hyperacute immune response after a MI is strongest during the sleep to wake transition period, making patients especially immunologically vulnerable at that time (Figure 7). A better understanding of the hyperacute immune response that occurs over a full 24-h period after an MI can provide new clues for the generation of improved therapies for MI.

## Data Availability

The raw data supporting the conclusion of this article will be made available by the authors, without undue reservation.

## Glossary

**ANOVA**: Analysis of variance
**APC**: Allophycocyanin
**Bmal1**: Brain and Muscle ARNT-Like
**CD**: Cluster of differentiation
**Clock**: Clock Circadian Regulator
**Cry2**: Cryptochrome Circadian Regulator 2
**CXCL**: C-X-C Motif Chemokine Ligand
**EDTA**: Ethylenediamine tetraacetic acid
**FBS**: Fetal bovine serum
**GM-CSF**: Granulocyte-macrophage colony-stimulating factor
**Icam1**: Intercellular Adhesion Molecule 1
**IP-10**: Interferon gamma-induced protein 10
**LAD**: Left anterior descending
**LSD**: Least significant difference
**Ly6G**: Lymphocyte antigen 6 complex locus G6D
**MI**: Myocardial infarction
**PBS**: Phosphate buffered saline
**PCI**: Percutaneous coronary intervention
**PE**: Phycoerythrin
**PE-Cy7**: PE-Cyanine7
**Per1**: Period Circadian Regulator 1
***Rorα***: Retinoic acid-related orphan receptor α
**Rplp0**: Ribosomal Protein Lateral Stalk Subunit P0
**RT-qPCR**: Real-time quantitative polymerase chain reaction
**SCN**: Suprachiasmatic nucleus
**SEM**: Standard error of the mean
**Sirt1**: Silent mating type information regulation 2 homolog 1
**T_C_-cells**: T-cytotoxic cells
**T_H_-cells**: T-helper-cells
**Vcam1**: Vascular cell adhesion protein 1
**ZT**: Zeitgeber Time
