# Supplementary material for: Circadian Dependence of the Acute Immune Response to Myocardial Infarction
Source: Front Pharmacol. 2022 May 25;13:869512. doi: 10.3389/fphar.2022.869512 (PMC9174900; doi:10.3389/fphar.2022.869512)
Supplement: Supplementary file 1 [file DataSheet1.PDF]

Supplementary Material

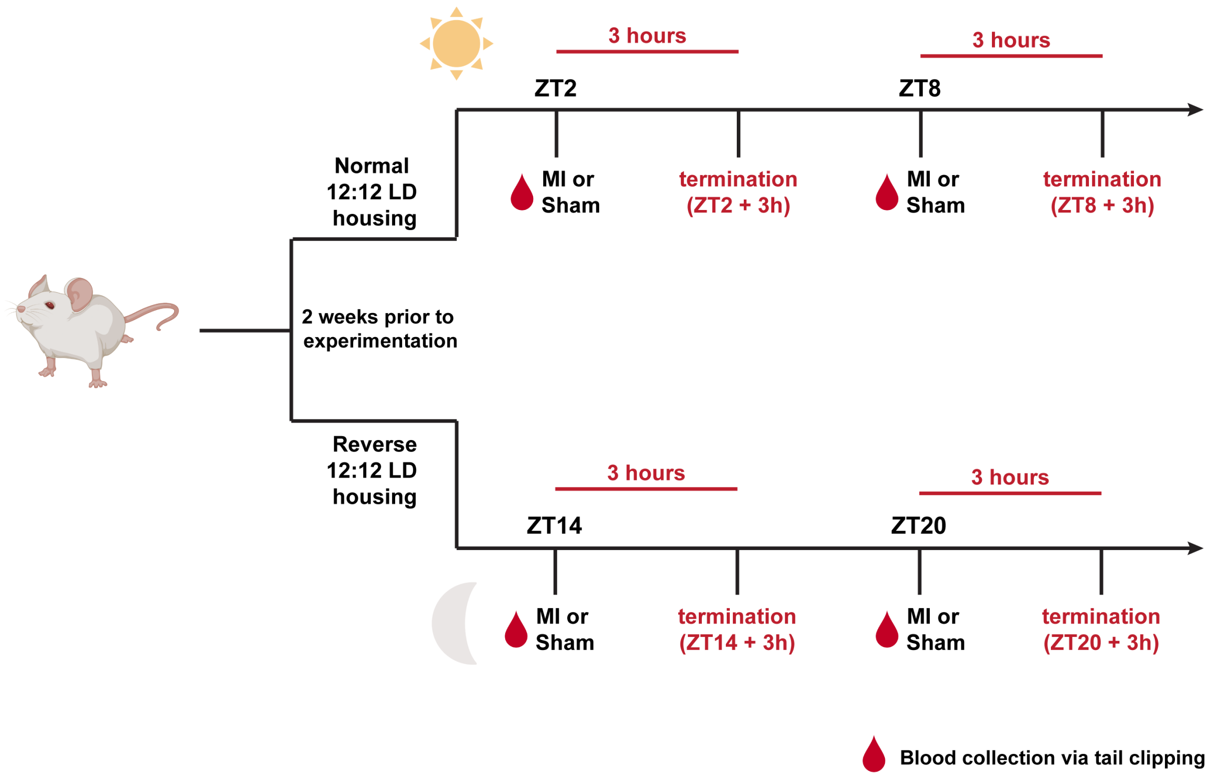

Supplementary Figure 1 – Graphic illustration of the experimental design. Balb/c mice aged 10-12 weeks were housed in a 12-hour light/12-hour dark cycle (lights on at ZT0, lights off at ZT12) for 2 weeks to allow synchronization of their circadian rhythms. Mice were subjected to myocardial infarction, by ligation of the left-descending coronary artery (LAD), or to sham surgery (without LAD ligation), at 4 different timepoints (ZT2, ZT8, ZT14 and ZT20). Baseline blood samples were collected via tail clipping prior to the start of the surgery. Three hours after surgery, animals were euthanized and their blood (via retro-orbital sinus) and hearts were collected.

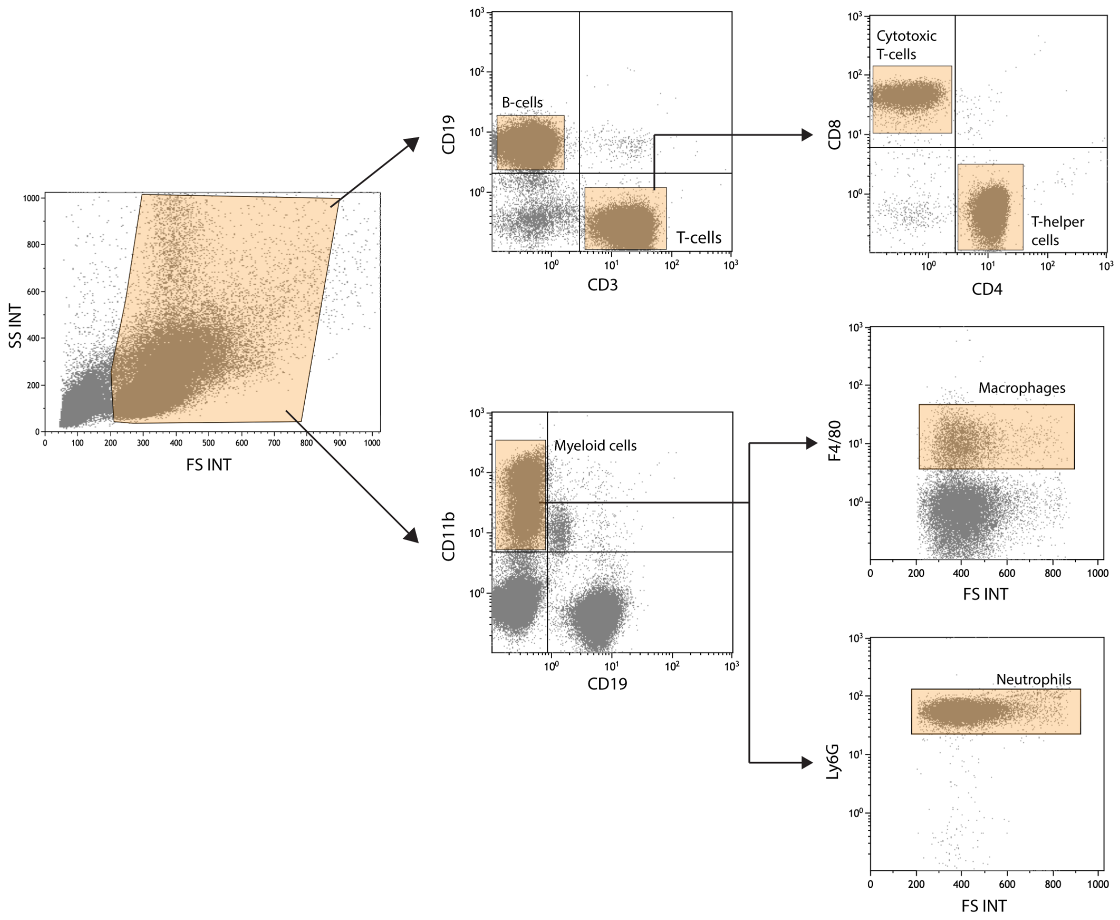

Supplementary Figure 2 – Representative plots demonstrating the discrimination of the different immune subsets.

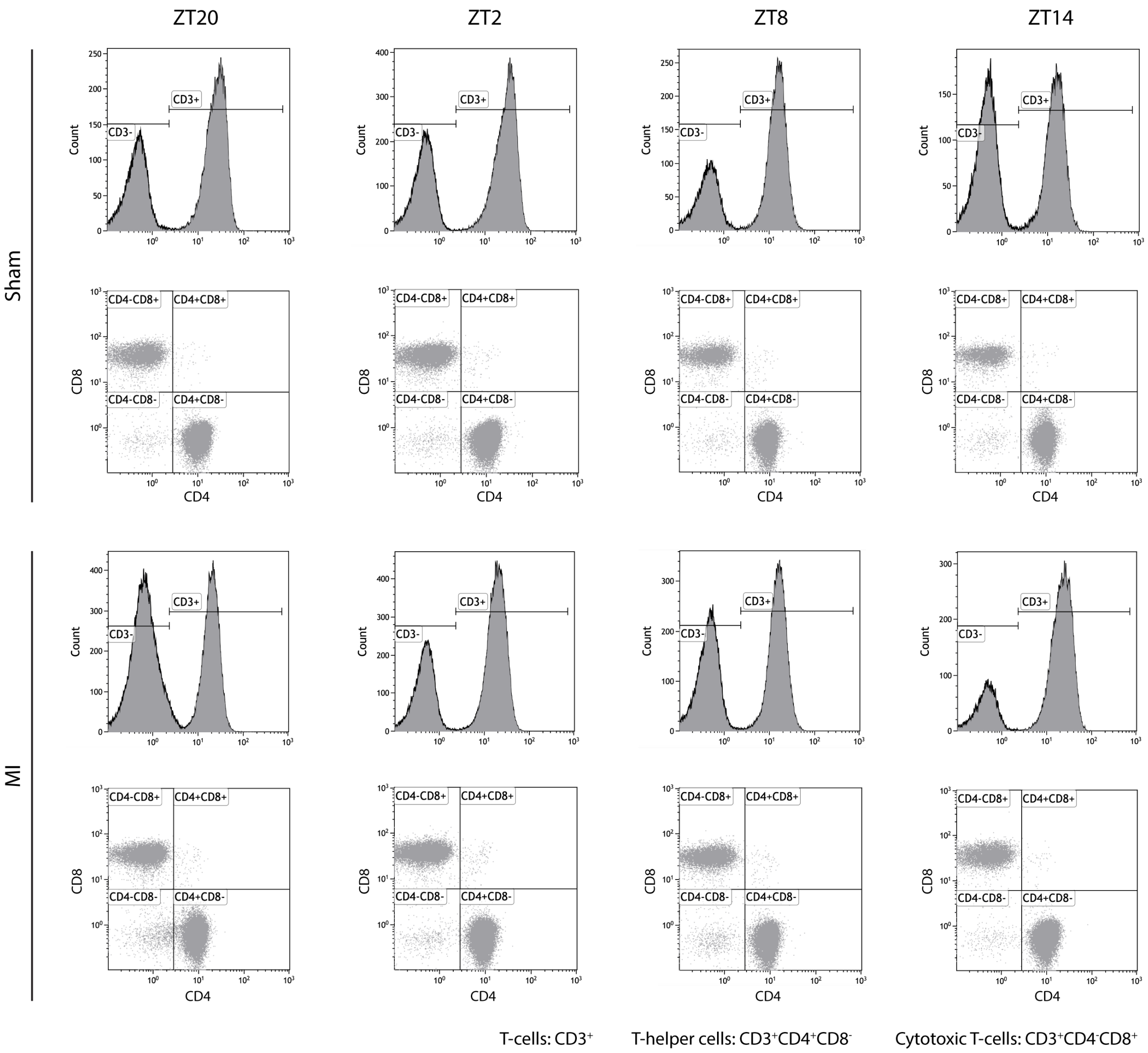

Supplementary Figure 3 – Representative plots showing the dynamics of blood circulating $CD3^{+}$ T-cells, $CD3^{+}CD4^{+}CD8^{-}$ T-helper cells and $CD3^{+}CD4^{-}CD8^{+}$ cytotoxic T-cells 3 hours after sham or MI surgery at ZT2, ZT8, ZT14 and ZT20.

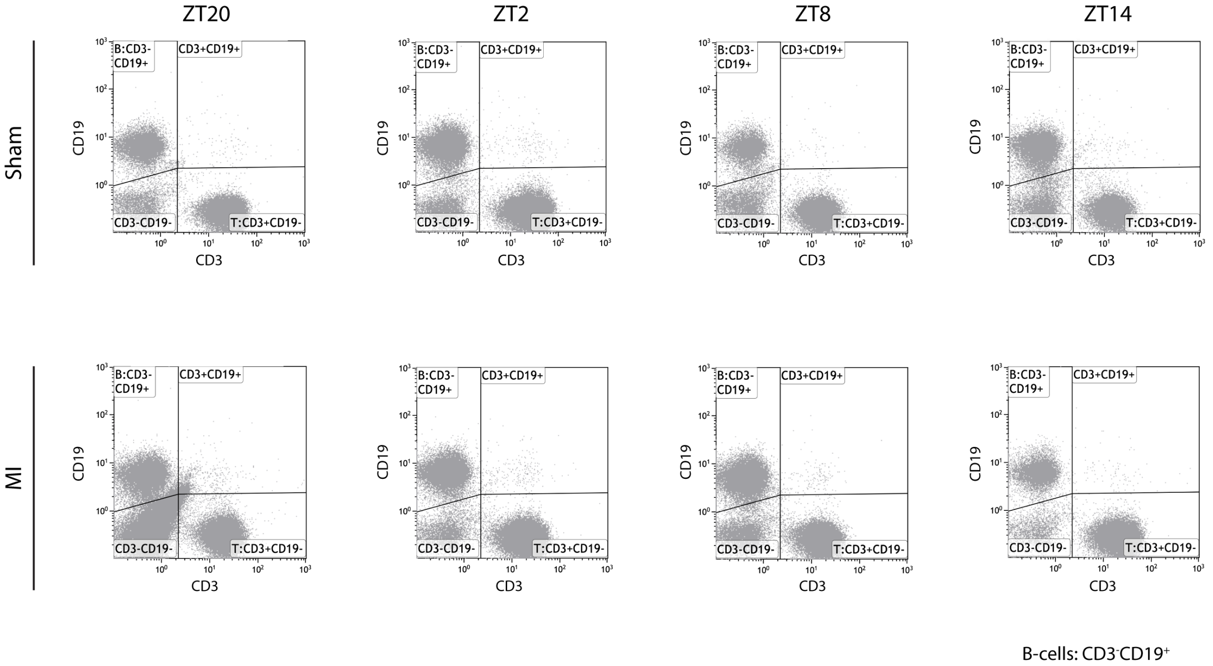

Supplementary Figure 4 – Representative plots showing the dynamics of blood circulating CD3$^-$CD19$^+$ B-cells 3 hours after sham or MI surgery at ZT2, ZT8, ZT14 and ZT20.

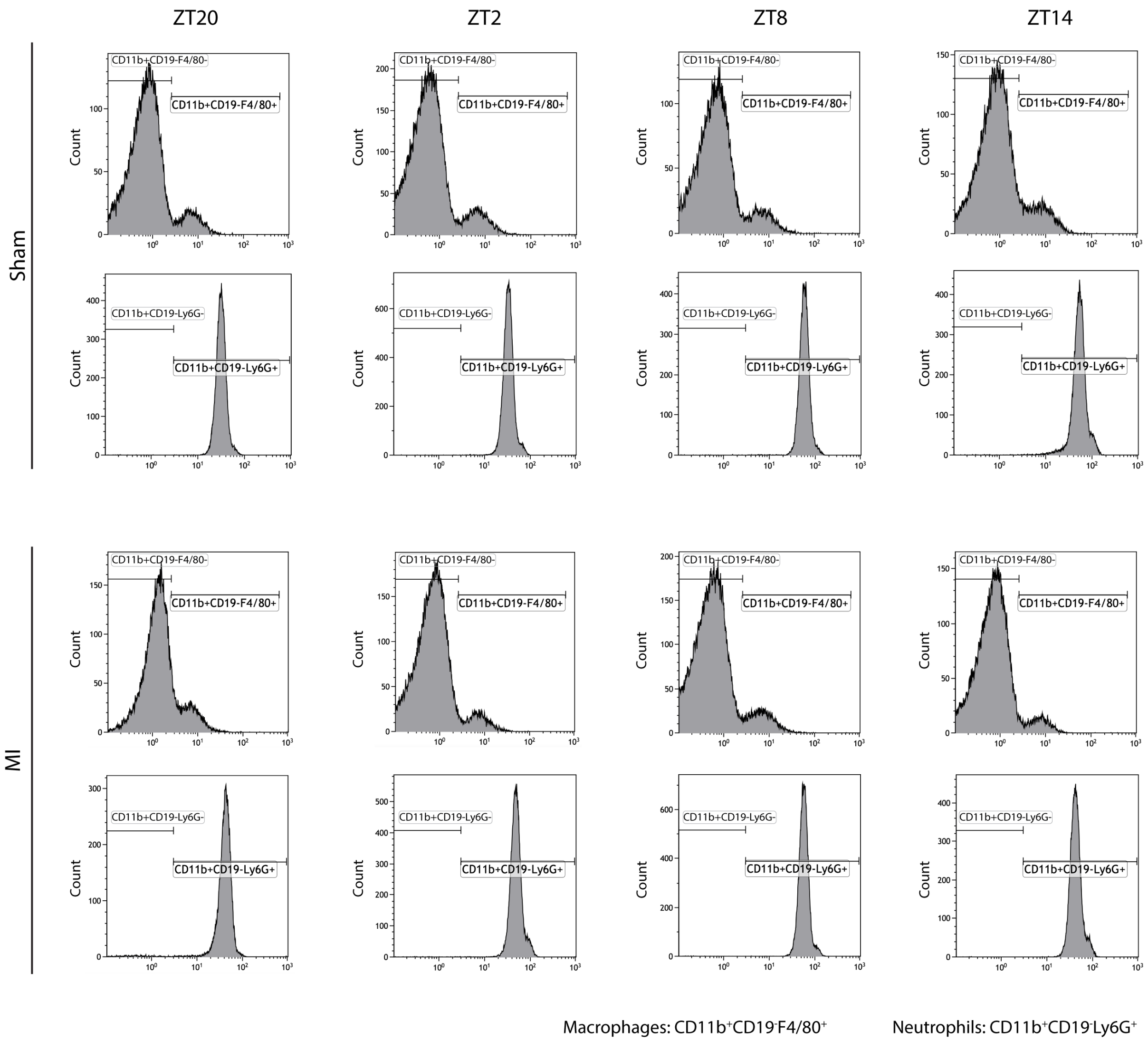

Supplementary Figure 5 – Representative plots showing the dynamics of blood circulating CD11b$^+$CD19$^-$F4/80$^+$ macrophages and CD11b$^+$CD19$^-$Ly6G$^+$ neutrophils 3 hours after sham or MI surgery at ZT2, ZT8, ZT14 and ZT20.

Supplementary Table 1 – Phase and amplitude of gene expression.

Gene	Amplitude		Acrophase		Bathyphase		Mesor	
	Sham	MI	Sham	MI	Sham	MI	Sham	MI
Vcam1	1,1978444	0,93084266	8,2632867	7,77074997	20.26328666000	19.77074997000	5,42875	5,65833
Cxcl2	0,7766962	0,77368392	10.95363240000	9,05850535	22.95363240000	21.05850535000	7,08083	6,71125
Cxcl1	0,4582091	0,19021187	7,4422743	9,62128724	19.44227426000	21.62128724000	7,0425	6,81
Icam1	0,311699	0,60280882	8,3989968	8,54574066	20.39899682000	20.54574066000	7,23208	7,19542
Bmal1	1.87710807000	2.06739537000	9.66861245000	9.92321538000	21.66861245000	21.92321538000	6.64583	6.28250
Per1	0.54067409000	0.94532218000	22.47801001000	23.77921088000	10.47801001000	11.77921088000	4.61292	4.82375
Cry2	1.16926288000	1.30764685000	22.72063099000	23.30671249000	10.72063099000	11.30671249000	6.52042	6.20583
Clock	0.67490380000	0.60707232000	7.11000708000	7.48987904000	19.11000708000	19.48987904000	4.03042	3.80708
Rev-Erbα	1.25585988000	0.77596222000	17.81445351000	18.49705778000	5.81445351000	6.49705778000	4.80458	4.87125
Rorα	1.41390810000	1.39000050000	4.59490013000	4.17423045000	16.59490013000	16.17423045000	11.05250	10.48917
Sirt1	1.33781798000	0.79347381000	10.93269915000	16.81554744000	22.93269915000	4.81554744000	6.65667	6.49375
